# Less surgical site infections in neurosurgery during COVID-19 times—one potential benefit of the pandemic?

**DOI:** 10.1007/s10143-021-01513-5

**Published:** 2021-03-05

**Authors:** T. Chacón-Quesada, V. Rohde, C. von der Brelie

**Affiliations:** Department of Neurosurgery, Georg August University of Göttingen, Göttingen, Germany

**Keywords:** Neurosurgery, COVID-19, Pandemic, Surgical site infection

## Abstract

Hygiene measures were intensified when the COVID-19 pandemic began. Patient contacts were limited to a minimum. Visitors were either not allowed for a certain period or limited for the rest of the time. The hospital staff began to wear masks and gloves continuously. Clinical examinations and routine wound controls were also performed under intensified hygiene standards. These circumstances result in a limitation of direct physical interactions between the nursing staff, the physicians and the patients. We analyzed to what extent the intensification of hygiene measures affects the rate of surgical site infections (SSI) after neurosurgical procedures. The rate of SSI during the 6-month interval after the beginning of COVID-19 measures was compared with the SSI rate before. The numbers of the period before COVID-19 were analyzed as mean values resulting from the analysis of two separate time periods each consisting of 6 months. The spectrum of surgical procedures was compared. Patient-related risk factors for SSIs were noted. Microorganisms were analyzed. We focused on SSIs occurring at a maximum of 60 days after the primary surgery. Overall, in the two respective 6-month periods before COVID-19, a mean of 1379 patients was surgically treated in our institution. After the beginning of COVID-19 (starting from 04/2020) our surgical numbers dropped by 101, resulting in a total number of 1278 patients being operated after 03/2020 until 09/2020. The SSI rate was 3.6% (03/2019–09/2019, 50 SSIs) and 2.2% (09/2019–03/2020, 29 SSIs), resulting in a mean of 2.9% before COVID-19 began. After the beginning of COVID-19 hygiene measures, this rate dropped to 1.4% (16 SSIs) resembling a significant reduction (*p*=0.003). Risk factors for the development of SSI were present in 81.3% of all patients. Pre- and post-COVID-19 patient groups had similar baseline characteristics. The same holds true when comparing the percentage of cranial and spinal procedures pre- and post-COVID-19 (*p*=0.91). Comparing the numbers (*p*=0.28) and the species (*p*=0.85) of microorganisms (MO) causing SSI, we found a similar distribution. Despite equal demographics and characteristics of SSI, the rate of SSI dropped substantially. This argues for an effective reduction of postoperative SSI resulting from the implementation of strict hygiene measures being established after the beginning of the COVID-19 pandemic. We therefore advocate continuing with strict and intensive hygiene measures in the future.

## Introduction

The COVID-19 pandemic has affected either directly or indirectly all medical fields. It has also exercised a huge influence on neurosurgery. The admission rate of patients was significantly affected [7]. Furthermore, certain types of elective neurosurgical procedures like transnasal surgeries, which were considered to be high-risk procedures for Sars-Cov2 transmission, had to be cancelled unless the surgery had to be indicated because of a neurological deficit. Despite the transmission and infection risks, neurosurgical procedures were performed in large numbers also during the pandemic. Sadly, our devoted neurosurgical care leads to the fact that some of our neurosurgical colleagues died due to the pandemic [1].

Prohibition of virus transmission is a key factor when it comes to measures curtailing the virus spread and “flattening the curve.” Thus, according to the local institutional guidelines, hygiene and public health measures have been significantly intensified to both protect patients and health workers in every health care institution. The beginning of these hygiene protocol changes was in March 2020 in Germany. Visitors were either not allowed for a certain period or limited for the rest of the time. The hospital staff began to wear masks and gloves continuously. Clinical examinations and routine wound controls were also performed under intensified hygiene standards. These circumstances resulted in a limitation of direct physical interactions between the nursing staff, physicians and patients. Postoperative surgical site infections (SSI) following cranial and spinal surgery are dreaded complications affecting the postoperative outcome, influencing morbidity and mortality and significantly reduce the patients´ life quality [4, 9–11]. Prevalences of SSIs in neurosurgery range from 0.5% in elective cranial or spinal surgery up to 20% or more in revision cases or after open or penetrating traumatic brain injury [2, 5].

The goal of the present study was to analyze the rate of SSIs after the beginning of COVID-19 hygiene measures.

## Methods

We performed a retrospective observational analysis of all SSIs treated in our department in adult patients since the instauration of the hygiene measurements related to the lockdown due to the COVID-19 pandemic from April until the end of September 2020. This analysis was carried out respecting the guidelines of good clinical practice following institutional ethical guidelines.

The main changes of the hygiene protocol are highlighted in Table 1.

**Table 1:** Key hygiene measures implemented in our institution

Continuous use of disposable surgical masks or FFP-2 masks by all staff, visitors.	
Visit restriction by non-medical/care-associated personnel (family members and other visitors)	
Contact reduction with medical and non-medical staff (medical students, interns, observers)	
More frequent and exhaustive cleaning protocols of rooms and surfaces	

Infections treated 12 months prior were also analyzed and divided into two groups both resembling 6-month time intervals (March 2019–August 2019, September 2019–February 2020). The data of the period before COVID-19 was analyzed as mean values resulting from the examination of these two separate time periods. Clinical data and demographics were recollected in order to compare not only infection rates but also clinical characteristics among the groups. For this part of the analysis, SSIs that developed more than 60 days after the initial surgery were excluded to focus on infections related to postoperative wound care and the implementation of COVID-19 hygiene measures by the hospital staff and exclude possible cofounders or causes out of the scope of our research. Wound revisions after surgeries being performed in other hospitals were not included. In purpose to reduce a potential confounding factor resulting from intrinsically infectious pathologies (brain abscesses, spondylodiscitis, other primary infectious diseases or surgery for open-traumatic brain injury), all of those cases are excluded, no matter if they were performed at our institution or not.

Patient demographics included age, sex and the pre-existence of patient-related risk factors for SSI. Patient-related risk factors considered were obesity, smoking, diabetes mellitus and vascular diseases. Other known risk factors analyzed were history of multiple interventions in the same surgical site, radiation and cerebrospinal fluid leak after the initial surgery. Numbers, multiplicity and type of pathogenic microorganisms (MOs) were analyzed. Surgeries were categorized in spinal or cranial procedures. Infections were classified in deep or superficial according to bone involvement. Information regarding an initial conservative treatment through antibiotic therapy was also recollected.

SPSS software (IBM Corp., Armonk, NY, USA) was used for statistical analysis. Continuous variables are presented as mean values and standard deviation. Categorical variables are depicted as numbers and percentages each. Categorical variables were compared in a cross-table fashion; statistical differences for categorical variables were calculated using chi-square test. After testing for normality, we applied a *t* test for statistical significance for continuous variables. A *p* value of < 0.05 was considered to represent significant differences.

## Results

Overall, in the first respective 6-months period before COVID-19, 1377 patients were surgically treated in our institution. In the second time interval, 1381 patients underwent surgery, resulting in a mean of 1379 patients. After the instauration of the COVID-19 measures, our surgical numbers decreased by 101. The SSI rate was 3.6% (03/2019–09/2019, 50 SSIs) and 2.2% (09/2019–03/2020, 29 SSIs), resulting in a mean of 2.9% before COVID-19 began. After the beginning of COVID-19 hygiene measures, this rate dropped to 1.4% (16 SSIs). This resembles a significant reduction of SSI rates (*p*=0.003).

Risk factors for the development of SSI were present in 81.3% of all patients. Comparison of pre- and post-COVID-19 groups revealed no differences (see Table 1). The site of SSIs did not differ regarding cranial and spinal procedures (*p=*0.91).

Comparing the number (*p=*0.28) and the species (*p*=0.85) of microorganisms (MO) causing SSI, we found a similar distribution between the cohorts pre- and post-COVID-19 (see Tables 2 and 3).

**Table 2:** Clinical and demographic characteristics of patients with SSI

Factor		Before COVID-19	During COVID-19	*p* value
Age (mean, SD)		66 (15.9)	62 (15.2)	0.44
Sex	M	29 (49.2%)	8 (50%)	N/A
	F	30 (50.2%)	8 (50%)	
Type of procedure	Cranial	36 (61%)	10 (62.5%)	0.91
	Spinal	23 (39%)	6 (37.5%)	
Preexisting patient-related risk factors	Yes	47 (79.7%)	14 (87.5%)	0.48
	No	12 (20.3%)	2 (12.5%)	
Time span to onset of SSI (SD)		30 (14.3)	27 (13.1)	0.45
Multiple surgeries?	Yes	11 (18.6%)	5 (31.2%)	0.27
	No	48 (81.4%)	11 (68.8%)	
Post surgical radiation	Yes	4 (6.8%)	2 (12.5%)	0.45
	No	55 (93.2%)	14 (87.5%)	
Post surgical CSF leakage	Yes	3 (5.1%)	2 (12.5%)	0,29
	No	56 (94.9%)	14 (87.5%)	
Pre-revision conservative antibiotic therapy	Yes	15 (25.4%)	2 (12.5%)	0.23
	No	44 (74.6%)	14 (87.5%)	
Type of infection	Superficial	20 (33.9%)	8 (50%)	0.24
	Deep	39 (66.1%)	8 (50%)

**Table 3:** Microbiology

Factor		Before COVID-19	Cranial	Spinal	During COVID-19	Cranial	Spinal	*p* value
Single or multiple MO?	No MO detected	13 (22.0%)	5	8	5 (29.4%)	1	4	0.38
	Single MO	32 (54.2%)	21	11	6 (35.3%)	4	2	
	Multiple MOs	14 (23.7%)	10	4	6 (35.3%)	5	1	
Detected pathogens	Staphylococci	39	26	13	10	7	3	0.85
	Other Gram-positive bacteria	11	8	3	4	4	0	
	Gram-negative Bacteria	12	9	3	4	3	1	

## Discussion

### Main results

Our analysis showed that the rate of SSI dropped dramatically since the beginning of the COVID-19 pandemic. This result was highly significant. In general, we were not surprised by the result that the rate of SSIs dropped since it seems logical that an increase in hygiene measures would affect the rate of postoperative SSIs, but the amplitude of the change is dramatic. No other measure in the last decades was able to create such a dramatic drop in SSI rate.

Overall numbers of surgical procedures only changed a little. Nonetheless, the numbers of procedures with a relatively low risk of SSIs (facet joint infiltrations, thermocoagulations of facet joint or iliosacral joint) were even performed less often after COVID-19. Moreover, the time interval after the beginning of the pandemic contains the warmer months of the year. Still, SSIs are considered to be higher in the warmer months of the year [3]. Both of these aspects result in a relatively (potentially false) high SSI rate after COVID-19 in our study.

The patients in the pre-COVID-19 era were comparable to the patients who underwent surgery during the COVID-19 pandemic. Risk factors for SSI development were also equally distributed among the groups. We also did not expect a change in the pathogenic MOs resulting in SSI. Our data confirmed this expectation. Most of the general anesthetic and surgical procedures have not changed. The patients still underwent the basic SSI-prohibitive protocols like preoperative antiseptic skin cleaning, preoperative antibiotics, glycemic control, postoperative wound checks and eventual postoperative antibiotic treatment.

Nonetheless, as in many other institutions, our hygiene measures intensified in every different part of in-patient care as part of the public health response to the pandemic. The main aspects are full-protective personal equipment of all personnel in the OR. Induction of anesthesia was oftentimes done with the minimum personnel since in many surgeries the COVID-19 infection status was not completely worked up. Also, intermediate cleaning procedures in the OR were intensified. The staff being involved in the pre- and postoperative treatment also continuously wore masks, oftentimes gowns and gloves. Patients themselves were also aware of the importance of hand hygiene, masks and social distancing. Wound checks were regularly performed, but with full-protective equipment and most of the times by only one physician. To sum it up, physical contact between care personnel and the patients has been reduced to an absolute minimum. This also holds true for physicians, ergo- and speech-therapists as well as psychologists. In general, but with few exceptions, visitors were not allowed since the pandemic started. This de-facto isolation of the patients potentially also contributes to the drop in the SSI rate.

### Generalizability

As expected, other studies have shown similar results. Hussain et al. in a recent article have shown similar results in a cohort study including patients who underwent thoracic surgery [6]. The same trend was shown by Losurdo and colleagues, who reported a drop from 8.4 to 3.3% in a cohort of patients who were operated in a general surgical department [8].

### Limitations

Given our data, we can describe just an association between the COVID-19 hygiene measures. We can just speculate that the dramatic reduction in SSI is to be explained with the change of hygiene measures; nonetheless, this conclusion seems rational. Furthermore, we could not determine which part of the intensified hygiene measures is the most important change, although this would be very interesting in order to understand the impact of the different parts of the hygienic strategy. Our analysis only covers a 6-month period, which, as a result, may lead to an underrepresentation of the SSI rate.

## Conclusions

Despite equal demographics and characteristics of SSI, the rate of SSI dropped dramatically. This argues for an effective reduction of postoperative SSI resulting from the implementation of strict hygiene measures being established after the beginning of the COVID-19 pandemic. We therefore advocated continuing with the strict hygiene measures in the future.

## Data Availability

The datasets analyzed during the current study are available from the corresponding author on reasonable request. All data generated or analyzed during this study are included in this published article.
